# Blood-Derived Systemic Inflammation Markers and Risk of Nodal Failure in Stage Ia Non-Small Cell Lung Cancer: A Multicentric Study

**DOI:** 10.3390/jcm12154912

**Published:** 2023-07-26

**Authors:** Federico Tacconi, Giuseppe Mangiameli, Emanuele Voulaz, Alexandro Patirelis, Federica Carlea, Eleonora La Rocca, Alessandro Tamburrini, Gianluca Vanni, Vincenzo Ambrogi

**Affiliations:** 1 Thoracic Surgery and Breast Unit, Department of Surgical Sciences, Tor Vergata University Polyclinic, Viale Oxford 81, 00133 Rome, Italy; alexandro.patirelis@ptvonline.it (A.P.); federica.carlea@ptvonline.it (F.C.); eleonora.larocca@virgilio.it (E.L.R.); gianluca.vanni@ptvonline.it (G.V.); ambrogi@uniroma2.it (V.A.); 2 Division of Thoracic Surgery, IRCCS Humanitas Research Hospital, Via Manzoni 56, Rozzano, 20089 Milan, Italy; giuseppe.mangiameli@hunimed.eu (G.M.); emanuele.voulaz@humanitas.it (E.V.); 3 Department of Biomedical Sciences, Humanitas University, Via Rita Levi Montalcini 4, Pieve Emanuele, 20090 Milan, Italy; 4 Unit of Cardio-thoracic Surgery, Southampton General Hospital, Tremona Road, Southampton SO166YD, UK; alessandro.tamburrini@uhs.nhs.uk

**Keywords:** non-small cell lung cancer, lymph node, staging, video thoracoscopy, lobectomy, systemic inflammation

## Abstract

Background: Unexpected spread to regional lymph nodes can be found in up to 10% of patients with early stage non-small cell lung cancer (NSCLC), thereby affecting both prognosis and treatment. Given the known relation between systemic inflammation and tumor progression, we sought to evaluate whether blood-derived systemic inflammation markers might help to the predict nodal outcome in patients with stage Ia NSCLC. Methods: Preoperative levels of neutrophil-to-lymphocyte ratio (NLR), platelet-to-lymphocyte ratio (PLR), and systemic inflammation score (SII, platelets × NLR) were collected from 368 patients who underwent curative lung resection for NSCLC. After categorization, inflammatory markers were subjected to logistic regression and time-event analysis in order to find associations with occult nodal spread and postoperative nodal recurrence. Results: No inflammation marker was associated with the risk of occult nodal spread. SII showed a marginal effect on early nodal recurrence at a quasi-significant level (*p* = 0.065). However, patients with T1c tumors and elevated PLR and/or SII had significantly shorter times to nodal recurrence compared to T1a/T1b patients (*p* = 0.001), while patients with T1c and normal PLR/SII did not (*p* = 0.128). Conclusions: blood-derived inflammation markers had no value in the preoperative prediction of nodal status. Nevertheless, our results might suggest a modulating effect of platelet-derived inflammation markers on nodal progression after the resection of tumors larger than 2 cm.

## 1. Introduction

Regional lymph node spread plays a pivotal role in patients with non-small cell lung cancer (NSCLC), as it affects both prognosis and treatment plans. Although patients with radiological evidence of stage I lung cancer are normally not offered invasive mediastinal staging prior to curative-intent resection, mediastinal nodal disease is found in approximately 7.5% of the cases at the final pathology report [1]. Furthermore, tumor-positive lymph nodes at any level can even be found in up to 11% of patients with a low-risk pattern (that is, peripherally located tumors, 10 mm or less [2,3]). For these reasons, it is important to develop strategies to optimize the pretreatment assessment and early follow-up of nodal status in patients with NSCLC.

In the last decade, the role of blood-derived systemic inflammation markers has progressively gained importance and popularity in the field of surgical oncology, as an elevated level of such markers has been consistently associated with worse prognosis in several clinical scenarios, including lung cancer [4,5,6,7]. Several mechanisms may explain this association. In the first instance, excessive systemic inflammation produces a dysfunction in cell-mediated anti-cancer immunity, which may enhance tumor growth and the metastatization process. In addition, systemic inflammation is associated with a higher tumor burden, even at a subclinical level. Whichever of the above two mechanisms prevails, the net effect is that excessive systemic inflammation seems to be intimately linked with faster tumor progression. Nonetheless, the implementation of these concepts into the actual routine clinical practice is still debatable, and systemic inflammation status is not included in decisional algorithms for the most common solid tumors. In this study, we aimed to assess whether blood-derived markers of systemic inflammation might be implemented in the prediction of occult nodal spread and early nodal recurrence in patients with stage Ia (T1anyN0) NSCLC who underwent curative-intent surgical resection.

## 2. Materials and Methods

This study is a spinoff of a larger multicentric investigation regarding the impact of systemic inflammation on lung cancer outcomes, and it entails a retrospective analysis of a prospectively collected database. Two of the participating centers are located in Italy (Policlinico Tor Vergata in Rome, and Humanitas Institute in Milan) and have had an established scientific partnership for several years now. The third center (Southampton General Hospital) is a large university hospital located in the United Kingdom, which participated in this study in the context of an informal cooperation program. The Internal Review Board of the first author’s home institution waived the need for a formal authorization process, given the non-experimental nature of the study and the use of anonymous clinical data. All patients gave their informed written consent to the surgical procedure. The study was conducted with adherence to the Transparent Reporting of Multivariable Prediction Model for Individual Prognosis and Diagnosis criteria [8].

In this study, we included patients who underwent standard lobectomy with either lobe-specific mediastinal nodal sampling or systematic mediastinal nodal dissection for cT1(any)N0 invasive lung adenocarcinoma, presenting as solid or part-solid lesions. The study period was January 2016–December 2020. After the initial selection, patients were excluded if they had had recent (<3 months) extra-thoracic surgery, trauma, or any other acute event (e.g., infections) potentially causing transient changes in white cell counts. Also, patients were excluded if they had abscessed tumors, hematological disorders, a previous history of malignancy, and if they had received immunosuppressants during the study period.

For each patient, we evaluated the preoperative level of the following systemic inflammation markers: neutrophil-to-lymphocyte ratio (i.e., the neutrophil count divided by lymphocyte count, NLR), platelet-to-lymphocyte ratio (i.e., platelet count divided by lymphocyte count, PLR), and systemic inflammation index (SII, i.e., the product of platelet count by NLR). For analytic purposes, a dummy variable was also created in order to divide patients with no inflammatory marker elevation from those with at least one abnormal inflammation marker (INFL > 0).

Blood-derived systemic inflammation markers were categorized and analyzed in terms of prediction ability, alongside a list of other dichotomous variables including age (cutoff: 70 years), gender, smoking status (current/former vs. never smoker), T1 subclass, categorized standard uptake value for ^18^F-fluorodeoxyglucose (SUV, cutoff > 5.0), and categorized comorbidity profile as expressed by the Charlson’s score (cutoff: >4). In order to adjust for the minimal technical variability related to the use of different blood counters at the participating centers, the blood-derived inflammation markers were categorized according to each center median. This method was also chosen because the receiver operating characteristic curves (ROC) did not show any reliable cutoff. Raw data are available upon request to the corresponding author. No author has any conflict of interest to disclose relevant to the present study.

### 2.1. Outcome Measures

All operated patients in which the final pathology showed unexpected hilar or mediastinal nodal involvement (hence depicting an upstaging scenario) and/or presented an isolated nodal recurrence within 24 months after lobectomy with pN0 status were defined as nodal failure cases. Patients who presented instead both nodal recurrence and extranodal progression (e.g., hilar stumps disease, contralateral or extrathoracic metastases) were considered nodal failure cases only if the nodal recurrence was the main feature of disease progression. Nodal failure was hence set at the primary outcome of the study. Secondary outcomes were overall survival and progression-free survival.

**Figure 1:**
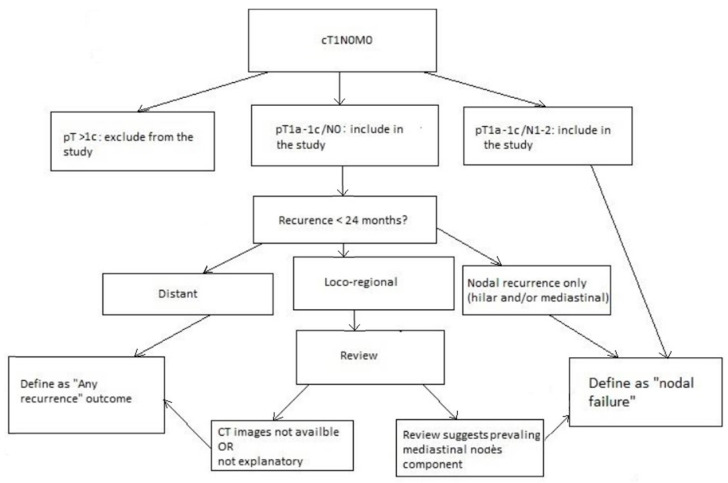
Study’s algorithm.

The flow-chart for patient selection and outcome definition is summarized in Figure 1.

### 2.2. Statistical Analysis

Differences among centers regarding basic patients’ characteristics were analyzed using the analysis of variance for continuous variables and the Pearson χ^2^ test for frequencies.

To explore the main working hypotheses of the study, we utilized a univariable logistic regression for each of the main outcomes. Factors presenting at least a non-significant association with the outcome of interest at *p* ≤ 0.1 were subsequently included in a parsimonious multivariable model, in order to test for independent prediction ability. Calibration of the regression models was assessed by means of the Homer–Lemeshow test, while discriminant ability was estimated by C-statistics. A stratified bootstrap analysis based on 1000 re-sampling cycles was applied in order to adjust for the potential bias due to the unbalanced sample sizes from participating centers. Listwise deletion was adopted to adjust for missing values.

For time-event analysis, curves were developed using the Kaplan–Meier method, and compared with the Log-Rank test. Comparison after stratification by inflammatory variables was also performed when appropriate. A *p*-value ≤ 0.05 was set as the significance threshold. Potential clinically relevant results with *p*-values between >0.05 and ≤0.1 were still emphasized and regarded as quasi-significant findings.

## 3. Results

The dataset included 368 patients. Table 1 reports the baseline patients’ data, and shows that the patients were relatively well-balanced among participating centers in terms of age, gender, smoking history and comorbidity profile. In one of the participating centers, there was a higher proportion of patients with T1a tumor, possibly as a result of a different regional policy in terms of secondary prevention strategies. A total of 206 patients received video-assisted lobectomy (VATS, either standard or uniportal), while the remaining 162 patients were operated on through a muscle-sparing thoracotomy. The median number of mediastinal lymph-nodes sampled during the operation was 7 (3), with no difference between centers (*p* = 0.248). Occult nodal spread was found in a total of 43 (11.6%) patients, with no difference between centers (*p* = 0.858). In particular, 24 patients (6.6%) were found to have mediastinal (pN2) spread (incidences per participating center: five (7.4%) at Policlinico Tor Vergata, two (3.7%) at Southampton Hospital, seventeen (6.8%) at Humanitas Institute, *p* = 0.667). Out of the 325 patients with initial pN0 staging, early nodal recurrence (any site) was seen in three cases at Center 1 (6.5%), one at Center 2 (2.0%), and nineteen (8.6%) at Center 3, respectively (*p* = 0.245).

### 3.1. Primary Outcomes

Univariable logistic regression results are summarized in Table 2, Table 3 and Table 4. As expected, the T1c subclass was significantly associated with a higher risk of occult nodal spread (*p* = 0.025), early nodal recurrence after initial pN0 staging (*p* = 0.048), and nodal failure (*p* = 0.004). The T1b subclass was significantly associated with a higher risk of nodal failure (*p* = 0.049). However, it was only marginally associated with the risk of occult nodal spread at a quasi-significant level (*p* = 0.053). Furthermore, no effect of the T1b subclass was seen on early recurrence after initial pN0 staging. Age > 70 years indicated a lower risk of occult nodal spread (*p* = 0.039). Nonetheless, this finding might mask a denominator bias due to less aggressive nodal dissection in older patients. An elevated standard-uptake value for ^18^F-fluorodeoxyglucose was marginally associated with occult nodal spread (*p* = 0.061). However, it was not associated with early nodal recurrence (*p* = 0.638), nor with nodal failure (*p* = 0.204). In general terms, the predictive performance of blood-derived systemic inflammation markers was poor. Indeed, none of these markers was clearly associated with higher risk of occult nodal spread (*p*-values for NRL, PRL and SII: 0.227, 0.374 and 0.764, respectively). However, elevated SII showed a marginal trend on the risk of early nodal recurrence (*p* = 0.065), while NLR was weakly associated with nodal failure (*p* = 0.078). No effect on any of the outcome variables was found for the participating center, smoking status, comorbidity profile, gender, and type of surgical access (VATS vs. open).

### 3.2. Multivariable Regression Results

Multivariable regression results are illustrated in Table 5. The T-subclass was strongly associated with a high risk of occult nodal spread (T1b vs. T1a: *p* = 0.025, T1c vs. T1a: *p* = 0.013), while marginal trends toward a higher risk were found for elevated SUV (*p* = 0.100) and younger age (*p* = 0.091). As far as mediastinal recurrence is concerned, no obvious independent predictor was identified. The T1c subclass was weakly associated with a higher risk (OR 2.71; CI 0.82–8.9), but statistical significance was not reached (*p* = 0.101). Elevated SII also did not go beyond a marginal association (HR 2.02, CI: 0.82–4.96; *p* = 0.124). As far as nodal failure is concerned, the T1c subclass was a strong independent predictor (*p* < 0.01), while—amongst inflammatory factors—only NRL showed a non-significant trend toward a higher risk (*p* = 0.082). The Homer–Lemeshow test showed that all three regression models had good calibration (*p*-values for occult nodal spread, early nodal recurrence and nodal failure: 0.672, 0.898, and 0.697, respectively). The discriminant ability evaluated by the area under the curve (AUC) of the predicted probabilities was fair with respect to occult nodal spread prediction (0.712), and poor with respect to the other two outcome measures (early recurrences: AUC: 0.612, nodal failure: AUC = 0.652).

### 3.3. Time-Event Analysis

Three-year overall and progression-free survival in the whole cohort were 88% and 75%, respectively. Our analysis showed that, among blood-derived inflammation markers, only elevated SII showed a non-significant trend toward early events (3-year survival rates: 86% vs. 90% in patients with normal SII, *p* = 0.101). The factors significantly associated with worse overall-survival were T-subclass (3-year survival rates: T1a 92%; T1b: 88%, T1c: 78%, *p* = 0.012), and Charlson comorbidity index > 4 (*p* = 0.050).

Amongst inflammatory variables, elevated NRL showed a non-significant trend towards a worse progression-free survival (3-year progression-free rate: 72% vs. 77% in patient with normal NRL, *p* = 0.086), while SII was significantly associated with a worse outcome (3-year progression-free rates: 70% vs. 79%, *p* = 0.036). Other factors that showed significant effects on progression-free survival were elevated SUV (69% vs. 79% 3-year survival rate, *p* = 0.030) and, again, T-subclass (3-year survival rates: T1a 88%; T1b: 71%; T1c 61%; *p* = 0.001).

Three-year nodal recurrence-free survival in patients with initial N0 status was 90%. No influence in this regard was reported for NLR (*p* = 0.246) and PLR (*p* = 0.358). Elevated SII showed a non-significant trend to higher risk nodal relapse (3-year nodal-recurrence free survival: 87% vs. 92%, *p* = 0.135). T-subclass was clearly associated with 3-year nodal recurrence-free survival rates: T1a: 95%, T1b: 91%, T1c: 71%, *p* = 0.001).

**Figure 2:**
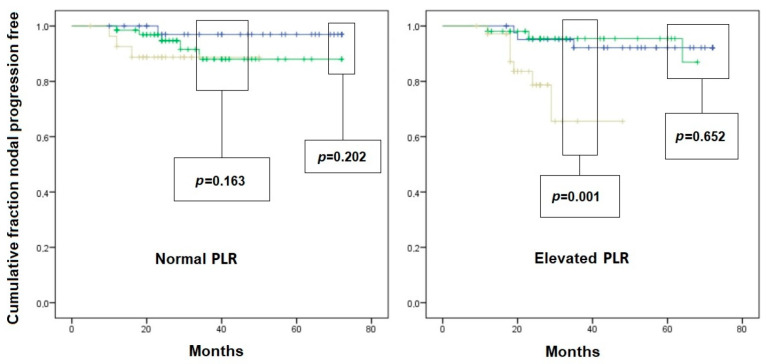
Kaplan–Meier curves of nodal-recurrence-free survival in patients with initial pN0 status. Left scheme: patient with normal platelet-to-lymphocyte ratio (PLR); right scheme: patients with elevated PLR. Blue line: pT1a subgroup; Green line: pT1b subgroup; Yellow line: pT1c subgroup. Comparison is given as pT1a vs. pT2b, and pT1c vs. pooled pT1a/pT1b survival, for graphical needs.

**Figure 3:**
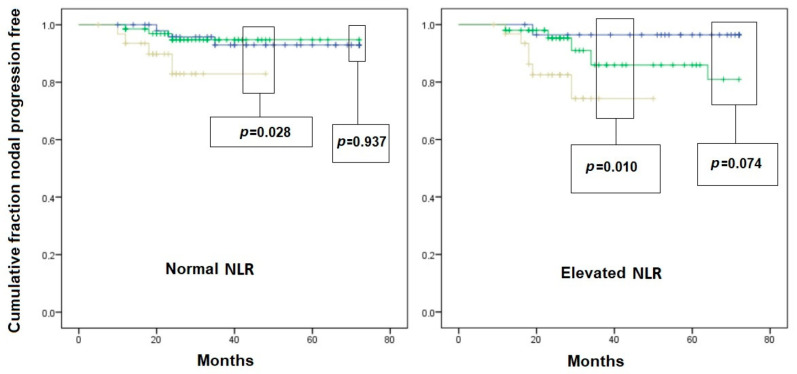
Kaplan–Meier curves of nodal-recurrence-free survival in patients with initial pN0 status. Left scheme: patient with normal Systemic Inflammation Index (SII); right scheme: patients with elevated SII. Blue line: pT1a subgroup; Green line: pT1b subgroup; Yellow line: pT1c subgroup. Comparison is given as pT1a vs. pT2b, and pT1c vs. pooled pT1a/pT1b survival, for graphical needs.

**Figure 4:**
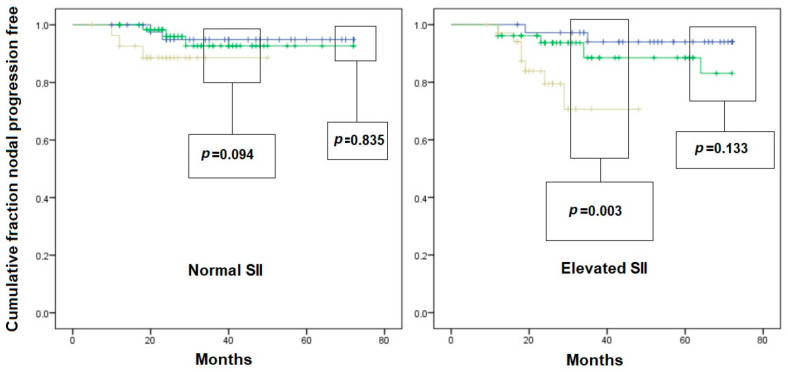
Kaplan–Meier curves of nodal-recurrence-free survival in patients with initial pN0 status. Left scheme: patient with normal neutrophil-to-lymphocyte ratio (NLR); right scheme: patients with elevated NLR. Blue line: pT1a subgroup; Green line: pT1b subgroup; Yellow line: pT1c subgroup. Comparison is given as pT1a vs. pT2b, and pT1c vs. pooled pT1a/pT1b survival, for graphical needs.

An interesting interaction was found when analyzing nodal recurrence-free survival curves after stratifying patients according to inflammatory variables. Indeed, in patients with normal PLR (Figure 2) and in those with normal SII (Figure 3), the T1c subclass did not result in a higher risk of nodal recurrence compared to T1a and T1b. On the other hand, in patients with elevated PLR and/or SII, there was a significantly faster nodal recurrence after resection of T1c tumors compared to T1a and T1b. In patients with elevated NLR (Figure 4), the difference in the nodal recurrence-free survival rate between the T1c and T1a/T1b subclasses became much more evident compared to the subgroup of patients with normal NRL. Furthermore, there was a trend toward a faster nodal recurrence in T1b patients compared with T1a, while this difference disappeared in patients with normal NLR.

## 4. Discussion

Adequate assessment of nodal involvement has a pivotal role in the management of patients with NSCLC. For this reason, invasive mediastinal staging is recommended for all patients at a higher risk of having nodal disease. High-risk patients are those with enlarged lymph nodes on imaging studies, and/or those with centrally located tumors with a diameter > 3 cm [9]. Patients with smaller primary tumors and no radiological evidence of enlarged nodes do not usually undergo further investigations before surgical treatment. Nonetheless, a remarkable percentage of these patients are eventually found to have unexpected hilar and/or mediastinal nodal involvement [1,2]. In this regard, methods to predict the presence of undetected nodal spread might help optimize decisional algorithms of treatment and postoperative follow-up of patients with NSCLC. This is particularly true in those circumstances when nodal assessment is incomplete, not reliable or not performed at all, as in the case of patients with early stage lung cancer treated with stereotactic body radiation therapy.

In recent years, there has been an increasing interest regarding the relevance of blood-derived inflammation markers within the field of clinical oncology. As a matter of fact, deranged values of a number of serum inflammatory markers and their ratios are generally associated with worse outcomes in several malignant disorders, including lung cancer. We therefore tried to assess whether certain commonly used inflammatory markers, either platelet- or neutrophil-based, could help to predict the nodal status and risk of nodal recurrence in patients with early stage NSCLC. We focused on these markers as they are routinely tested in standard clinical practice within the preoperative assessment and therefore immediately available essentially in all centers performing lung resections for cancer. Other serum markers such as Carcino-Embryonic Antigen (CEA)—which has also been associated with occult nodal spread in early stage NSCLC [1,10,11]—are not typically measured before surgical treatment of early NSLCL in many centers. Recently published literature on this topic also corroborated the basic idea of our study. For example, Qu et al. [12] found that a high platelet count is independently associated with nodal spread in patients with lung adenocarcinoma. An interesting cause–effect relationship was also hypothesized in the same study, with a focus on pro-metastastic activity of so-called tumor-educated platelets via the enhanced secretion of Vascular-Endothelial Growth Factor-A. Interestingly, these authors conceive a potential role of antiplatelet drugs as a protection against lymph-node spread in patients with NSCLC [12]. Duan and coworkers found that NLR is associated with overall tumor burden and extent of lymph-node invasion in resected esophageal cancer [13]. Zhang et al. [6] found that NLR affects survival figures and it is associated with nodal involvement in resected NSCLC. Also of interest, Resio et al. [14] found that obesity—a condition associated with chronic dysregulation of systemic inflammation—is a risk factor for occult nodal spread in patients with stage Ia/b NSCLC. All of these studies, however, did not focus specifically on patients with stage I lung cancer, while we aimed at assessing the potential implications of blood-derived systemic inflammatory markers on this precise subgroup of patients. Therefore, we tested this hypothesis against a series of other well-known risk factors and potential confounders. In order to capture any potential association, we also adopted a more liberal use of *p*-values.

In our study, SII and NLR, but not PLR, showed just a marginal predictive value on the outcomes of interest, when assessed in the context of a logistic regression analysis. In particular, NLR was marginally associated with the composite outcome nodal failure, while elevated SII only depicted a trend to higher risk of nodal recurrence at quasi-significant level (*p* = 0.065). This trend was even weaker when extending the analysis beyond postoperative month 24, as indicated by time-event analysis. Furthermore, no inflammation marker was associated with the risk of occult nodal spread found at the time of surgical operation.

We instead had different and more interesting results when exploring postoperative nodal recurrence in a time-event manner and within a stratified analysis. We found that an increase in any blood-derived inflammation marker significantly affected the nodal progression in patients with larger primary tumors (that is, T1c and at some extent, T1b). Based on these considerations, our impression is that, although deranged systemic inflammation fails as a predictor of occult nodal spread at the time of surgery, it might still play a role by enhancing local metastatization and recurrence in patients who already have an increased risk of nodal progression. It these patients, pre-existing derangement in systemic inflammation might add to the additional inflammatory changes caused by the surgical operation itself [15], hence increasing the postoperative anti-cancer immunodeficiency with a sort of double-hit mechanism. Although this model should only be considered as hypothetical, it might well explain why blood-derived inflammation markers showed some association with postoperative nodal failure, but not with the risk of occult nodal spread at the time of surgery. According to this model, the clinical relevance of factors modulating the extent of perioperative inflammation might be investigated in future studies. In this regard, we did not observe any differences in the main outcomes between patients who received either VATS or open surgery. Although this was not the primary focus of our study, a possible explanation of this finding is that patients in the “open surgery” group were actually operated through a limited muscle-sparing thoracotomy, with an extent of postoperative inflammatory changes almost comparable to VATS [15].

Based on most of the above-mentioned literature [6,12,13,14], the lack of a clear association between blood-derived systemic inflammation markers and occult nodal spread at the time of surgery was a rather unexpected finding, especially as far as platelet-derived indexes are concerned. However, it should be noted that platelet-derived systemic inflammation indexes showed no association with nodal status in some other studies as well [6,7]. These discrepancies might be explained by several factors, including (but not limited to) different cutoffs in use, inhomogeneous patient subgroups, and different setting of statistical regression models. Furthermore, complex functional interactions between circulating inflammatory cells may happen under certain circumstances, thus increasing the degree of variability of reported results. For example, Sulibhavi et al. [5] showed that isolated high lymphocyte count is associated with worse survival after major surgery for pT1 NSCLC. This finding seems in contradiction with the assumption of a protective role against tumor progression of circulating lymphocytes, and suggests that inflammatory prognostic markers utilizing the lymphocytes count should be used cautiously. The issue becomes even more complex when considering the interactions between systemic inflammation and local inflammatory events occurring at the tumor site. In this regard, one must consider that the so-called tumor-infiltrating immune cells are able to eliminate tumor cells, but also (paradoxically) to create a supportive micro-environment favoring their escape and metastatization [16].

Yet another finding that we consider worth discussing is the fact that, in our study, the primary tumor size > 2 cm (T1c) displayed a higher risk of occult nodal involvement. This finding was expected, and in keeping with some previous studies [17,18]. Conversely, other authors reported different results. Roy et al. [19] could not find any correlation between tumor size and risk of occult nodal spread in stage I NSCLC presenting part-solid radiological pattern. Similar considerations came from DuComb et al. [20], and Ghaly et al. [3]. Furthermore, in patients with stage I NSCLC treated with stereotactic body radiotherapy, the size of the tumor did not correlate with nodal failure [21]. A survey from the Italian VATS Group also found that neither SUV, nor T-subclass were associated with occult nodal spread [22]. Again, different designs of multivariable regression analyses might explain some of these discrepancies and the extent and variations of mediastinal nodal dissection may also act as a confounding factor.

Certain limitations of our study must be acknowledged. First, the multicentric design of it naturally causes some discrepancies in terms of basic characteristics of the patients. Furthermore, some variables are missing. For example, we could not include histology subtype, grading and location of the primary tumor (central vs. peripheral). However, we wanted to test the relevance of systemic inflammation indexes with a specific view to pre-treatment prediction, that is, in a phase when precise histology assessment might not be available in the real world scenario. Yet another limitation is that we could not include changes in circulating cells morphology, and just relied on absolute counts. Finally, different surgical groups performed the operations, a fact that might lead to different extents of nodal harvesting. However, two of the participating centers have a long-standing cooperation in place, so that they share common habits in terms of instruments use, surgical techniques and surgical viewpoints, thus making this possibility less probable. Furthermore, the co-author from the third center abroad (AT) had previous 2-year training at the principal investigator’s home institution. However, in order to adjust for any discrepancy in terms of nodal sampling, we purposefully included early nodal recurrence as a part of a composite endpoint. This was carried out to increase the chance of capturing cases of nodal failure due to pathological nodal tissue being left behind.

In conclusion, in this study we found that including a blood-derived systemic inflammation marker might help to identify a specific subgroup of patients with higher risk of nodal recurrence after major resection for pT1cN0 lung cancer. However, there seems to be no role of blood-derived systemic inflammation in predicting nodal status before surgery. Based on our results, the opportunity of a tailored follow-up plan in patients with elevated inflammation markers and primary tumor diameter > 20 mm should be explored in future studies. Further investigation on the possible role of antiplatelets and other anti-inflammatory drugs in this context would also be welcome.

## Figures and Tables

**Table 1:** Baseline findings.

	PTV (Center I)	SH (Center 2)	HI (Center 3)	*p*-Value
Patient n.	67	54	247	-
Age in years [IQR]	70.0 [64–76]	70.0 [65–78]	69.0 [61.5–76]	0.484
Male/Female (ratio)	39/28	26/28	121/126	0.393
Smoking status (count/percent)				
Never	13 (19.4%)	8 (14.8%)	45 (18.2%)	
Former	32 (47.7%)	33 (58.9%)	125 (50.6%)	0.654
Current	22 (32.8%)	13 (24.1%)	77 (31.1%)	
Charlson comorbidity index	5.2 (2)	4.9 (2.3)	4.8 (0.8)	0.762
T subclass (count/percent)				
T1a	5 (7.4%)	4 (7.4%)	84 (34.0%)	
T1b	34 (50.7%)	23(42.6%)	106 (42.9%)	<0.001
T1c	28 (41.8%)	27 (50.0%)	57 (23.1%)	
SUV > 5.0	32 (47.7%)	29 (53.7%)	110 (44.5%)	0.876
NLR [IQR]	2.4 [1.9–3.1]	2.2 [1.5–2.8]	2.8 [1.1–3.2]	0.064
PLR [IQR]	125.6 [89.9–150.5]	120.0 [93.4–145.0]	149.2 [93.7–190.2]	0.021
SII (1000/mm^3^) [IQR]	292.5 [153.4–440.8]	527.0 [336.6–720.6]	422.3 [156.2–720.3]	<0.001

Legend: HI: Humanitas Institute (participating Center 3); NLR: neutrophil-to-lymphocyte ratio; PLR: platelets-to-lymphocyte ratio; PTV: Policlinico Tor Vergata (participating Center 1); IQR: Interquartile Range; SII: systemic inflammation index; SH: Southampton Hospital (participating Center 3); SUV: standard uptake value for ^18^F-fluorodeoxyglucose. All continuous values are given as median (quartile range).

**Table 2:** Univariable logistic regression results. Outcome: occult nodal spread.

	OR	95% CI	*p*-Value
Age > 70 years	0.49	0.25–0.96	0.039
Female Sex	Reference	-	-
Male Sex	0.96	0.51–1.82	0.918
Center:			
PTV	Reference	-	-
SH	1.35	0.50–3.66	0.551
HI	1.33	0.40–4.32	0.634
VATS approach	Reference	-	-
Open approach	0.65	0.22–1.94	0.450
Never smoker	Reference	-	-
Former/current	0.77	0.42–1.27	0.364
Charlson’s score > 4	1.29	0.65–2.57	0.454
SUV > 5.0	2.17	0.98–4.47	0.061
T-class			
T1a	Reference	-	-
T1b	2.60	0.94–7.15	0.053
T1c	3.14	1.11–8.89	0.025
NLR	1.48	0.78–2.80	0.227
PLR	0.745	0.39–1.42	0.374
SII	0.90	0.47–1.72	0.764
INFL > 0	0.98	0.50–1.92	0.952

Legend: HI: Humanitas Institute (partecipating center 3; INFL > 0: at least one inflammation marker above median: NLR: neutrophil to lymphocyte ratio; PLR: platelet to lymphocyte ratio; PTV: Policlinico Tor Vergata (partecipating center 1); SII: systemic inflammation score; SH: Southampton Hospital (partecipating center 2); SUV: standard uptake value for ^18^F-fluorodeoxyglucose; VATS: video-assisted thoracic surgery.

**Table 3:** Univariable logistic regression results. Outcome: early nodal recurrence after initial N0 staging.

	OR	95% CI	*p*-Value
Age > 70 years	1.402	0.59–3.30	0.439
Female Sex	Reference	-	-
Male Sex	1.52	0.36–1.63	0.342
Center:			
PTV	Reference	-	-
SH	1.92	0.67–3.21	0.144
HI	1.76	0.75–2.75	0.303
VATS approach	Reference	-	-
Open approach	1.87	0.63–2.92	0.223
Never smoker	Reference	-	-
Former/current	0.72	0.38–1.36	0.317
Charlson’s score > 4	0.48	0.17–1.35	0.116
SUV > 5.0	0.76	0.25–2.35	0.638
T-class			
T1a	Reference	-	-
T1b	1.25	0.36–4.29	0.734
T1c	2.89	1.01–9.46	0.048
NLR	1.72	0.73–4.05	0.211
PLR	1.65	0.69–3.94	0.256
SII	2.19	0.90–5.34	0.065
INFL > 0	2.06	0.73–5.71	0.164

Legend: HI: Humanitas Institute (partecipating center 3; INFL > 0: at least one inflammation marker above median: NLR: neutrophil to lymphocyte ratio; PLR: platelet to lymphocyte ratio; PTV: Policlinico Tor Vergata (partecipating center 1); SII: systemic inflammation score; SH: Southampton Hospital (partecipating center 2); SUV: standard uptake value for ^18^F-fluorodeoxyglucose; VATS: video-assisted thoracic surgery.

**Table 4:** Univariable logistic regression results. Outcome: nodal failure.

	OR	95% CI	*p*-Value
Age > 70 years	0.76	0.44–1.31	0.317
Female Sex	Reference	-	-
Male Sex	1.19	0.69–2.03	0.522
Center:			
PTV	Reference	-	-
SH	1.00	0.48–2.07	1.000
HI	0.511	0.20–1.26	0.146
VATS approach	Reference	-	-
Open approach	1.14	0.51–2.54	0.737
Never smoker	Reference	-	-
Former/current	0.89	0.69–1.32	0.575
Charlson’s score > 4	0.93	0.52–1.67	0.812
SUV > 5.0	1.55	0.78–3.06	0.204
T-class			
T1a	Reference	-	-
T1b	2.06	1.07–4.58	0.049
T1c	3.13	1.47–7.46	0.004
NLR	1.62	0.94–2.78	0.078
PLR	1.04	0.61–1.79	0.867
SII	1.30	0.76–2.24	0.332
INFL > 0	1.32	0.74–2.36	0.337

Legend: HI: Humanitas Institute (partecipating center 3; INFL > 0: at least one inflammation marker above median: NLR: neutrophil to ly;mphocyte ratio; PLR: platelet to lymphocyte ratio; PTV: Policlinico Tor Vergata (partecipating center 1); SII: systemic inflammation score; SH: Southampton Hospital (partecipating center 2); SUV: standard uptake value for ^18^F-fluorodeoxyglucose; VATS: video-assisted thoracic surgery.

**Table 5:** Multivariable logistic regression results. Only significant or quasi-significant findings are reported.

	OR	95% CI	*p*-Value
**Outcome: occult nodal spread**			
T-subclass			
T1a	Reference	-	-
T1b	5.72	1.24–26.33	0.025
T1c	7.32	1.53–35.01	0.013
Age > 70 years	0.46	0.19–1.13	0.090
SUV > 5.0	2.02	0.87–4.71	0.100
**Outcome: early nodal recurrence**		No significant independent factor found	
**Outcome: nodal failure**			
NLR	2.41	0.85–7.02	0.082
T-subclass			
T1a	Reference	-	-
T1b	1.90	0.89–4.44	0.090
T1c	3.17	1.40–7.16	0.006

Legend: NLR: neutrophil-to-lympocyte ratio; SUV: standard uptake value for ^19^F-fluorodeoxyglucose.

## Data Availability

Raw data are available on SPSS/Excel format upon reasonable request to the first author.

## References

[1] Koike T., Koike T., Yamato Y., Yoshiya K., Toyabe S. (2012). Predictive risk factors for mediastinal lymph node metastasis in clinical stage IA non-small cell lung cancer patients. J. Thorac. Oncol..

[2] Deng H.Y., Zhou J., Wang R.L., Jiang R., Zhu D.X., Tang X.J., Zhou Q. (2020). Lobe-specific lymph node dissection for clinical early stage (cIA) peripheral non-small cell lung cancer patients: What and how?. Ann. Surg. Oncol..

[3] Ghaly G., Rahouma M., Kamel M.K., Nasar A., Harrison S., Nguyen A.B., Port J., Stiles B.M., Altorki N.K., Lee P.C. (2017). Clinical predictors of nodal metastases in peripherally clinical T1aN0 non-small cell lung cancer. Ann. Thorac. Surg..

[4] Wang J., Hui Z., Men Y., Kang J., Sun X., Deng L., Zhai Y., Wang W., Bi N., Liang J. (2019). Systemic inflammation-immune status predicts survival in stage III-N2 non small cell lung cancer. Ann. Thorac. Surg..

[5] Sulibhavi A., Asokan S., Miller M.I., Moreira P., Daly B.D., Fernando H.C., Litle V.R., Suzuki K. (2020). Peripheral blood lymphocytes and platelets are prognostic in surgical pT1 non-small cell lung cancer. Ann. Thorac. Surg..

[6] Zhang H., Xia H., Zhang L., Zhang B., Yue D., Wang C. (2015). Clinical significance of preoperative neutrophil-lymphocyte vs platelet-lymphocyte ratio in primary operable patients with non-small cell lung cancer. Am. J. Surg..

[7] Guo W., Cai S., Zhang F., Shao F., Zhang G., Zhou Y., Zhao L., Tan F., Gao S., He J. (2019). Systemic immune-inflammation index (SII) is useful to predict survival outcomes in patients with surgically resected non-small cell lung cancer. Thorac. Cancer.

[8] Collins G.S., Reitsma J.B., Altman D.G., Moons K.G. (2015). Transparent Reporting of a multivariable prediction model for Individual Prognosis Or Diagnosis (TRIPOD): The TRIPOD Statement. Ann. Int. Med..

[9] De Leyn P., Dooms C., Kuzdzal J., Lardinois D., Passlick B., Rami-Porta R., Turna A., Van Schil P., Venuta F., Waller D. (2014). Revised ESTS guidelines for preoperative mediastinal lymph node staging for non-small cell lung cancer. Eur. J. Cardio Thorac. Surg..

[10] Zhang C., Pang G., Ma C., Wu J., Wang P., Wang K. (2019). Preoperative Risk Assessment of Lymph Node Metastasis in cT1 Lung Cancer: A Retrospective Study from Eastern China. J. Immunol. Res..

[11] Nasralla A., Lee J., Dang J., Turner S. (2020). Elevated preoperative CEA is associated with subclinical nodal involvement and worse survival in stage I non-small cell lung cancer: A systematic review and meta-analysis. J. Cardiothorac. Surg..

[12] Qu C.H., Li T., Tang Z.P., Zhu X.R., Han J.Y., Tian H. (2020). Platelet count is associated with the rate of lymph node metastasis in lung adenocarcinoma. Cancer Manag. Res..

[13] Duan H., Zhang X., Wang F.-X., Cai M.-Y., Ma G.-W., Yang H., Fu J.-H., Tan Z.-H., Meng Y.-Q., Fu X.-Y. (2015). Prognostic role of neutrophil-lymphocyte ratio in operable esophageal squamous cell carcinoma. World J. Gastroenetrol..

[14] Resio B.J., Canavan M., Mase V., Dhanasopon A.P., Blasberg J.D., Boffa D.J. (2020). Invasive staging procedures do not prevent nodal metastases from being missed in stage I lung cancer. Ann. Thorac. Surg..

[15] Tacconi F., Carlea F., La Rocca E., Ambrogi V. (2000). Systemic inflammation after Uniport, Multiport, or Hybrid VATS lobectomy for lung cancer. Thorac. Cardiovasc. Surg..

[16] Bremnes R.M., Al-Shibli K., Donnem T., Sirera R., Al-Saad S., Andersen S., Stenvold H., Camps C., Busund L.-T. (2011). The role of tumor-infiltrating immune cells and chronic inflammation at the tumor site on cancer development, progression, and prognosis: Emphasis on non-small lung cancer. J. Thorac. Oncol..

[17] Lee P.C., Port J.L., Korst R.J., Liss Y., Meherally D.N., Altorki N.K. (2007). Risk factors for occult mediastinal metastases in clinical stage I non-small cell lung cancer. Ann. Thorac. Surg..

[18] Lee S.Y., Jeon J.H., Jung W., Chae M., Hwang W.J., Hwang Y., Cho S., Chung J.-H., Kim K., Jheon S. (2021). Predictive factors for lymph node metastasis in clinical stage I part-solid lung adenocarcinoma. Ann. Thorac. Surg..

[19] Roy P., Lévesque-Laplante A., Guinde J., Lacasse Y., Fortin M. (2020). Central tumor location and occult lymph node metastasis in cT1N0M0 non-small cell lung cancer. Ann. Am. Thorac. Soc..

[20] DuComb E.A., Tonelli B.A., Tuo Y., Cole B.F., Mori V., Bates J.H., Washko G.R., Estépar R.S.J., Kinsey C.M. (2020). Evidence for expanding invasive mediastinal staging for peripheral T1 lung tumors. Chest.

[21] Marhawa G., Stephans K.L., Woody N.M., Reddy C.A., Videtic G.M. (2014). Lung sterotactic body raditation therapy: Regional nodal failure is not predicted by tumor size. J. Thorac. Oncol..

[22] Marulli G., Faccioli E., Mammana M., Nicotra S., Comacchio G., Verderi E., De Palma A., Rea F., Italian VATS Group (2020). Predictors of nodal upstaging in patients with cT1-T3N0 non’-small cell lung cancer (NSCLC): Results from the Italian VAS Group Registry. Surg. Today.

